# Combining enzalutamide with PARP inhibitors: Pharmaceutically induced BRCAness

**DOI:** 10.18632/oncotarget.22074

**Published:** 2017-10-25

**Authors:** Timothy C. Thompson, Likun Li, Bradley M. Broom

**Affiliations:** Timothy C. Thompson: Department of Genitourinary Medical Oncology, Division of Cancer Medicine, The University of Texas MD Anderson Cancer Center, Houston, TX, USA

**Keywords:** castration-resistant prostate cancer, enzalutamide, PARP inhibitors, combination therapy

Androgen deprivation therapy (ADT) has been the standard of care for advanced prostate cancer (PCa) for decades, yet men with this condition inexorably develop resistance to ADT and progress to metastatic castration-resistant PCa (mCRPC) [1]. Second generation androgen receptor (AR) signaling inhibitors such as enzalutamide (ENZ) can prolong survival in mCRPC patients, however, ENZ-resistance almost invariably arises and the disease continues to progress. Recently, poly(ADP-ribose) polymerase (PARP) inhibition (olaparib, OLA) was shown to prolong survival in mCRPC patients who had received prior treatment with a second generation AR signaling inhibitor, and whose tumors harbored mutations in specific DNA repair genes, including BRCA2 and ATM [2]. Unfortunately, as with ENZ, resistance to PARP inhibition arises, and progression resumes. To optimize ENZ and PARP inhibitor combination therapy, we pursued efficacy and mechanistic studies using CRPC preclinical models.

It was shown previously that AR transcriptionally upregulates multiple DNA damage response (DDR) genes [3-5], and that DDR genes are upregulated in PCa metastases [5]. Interestingly, PARP activities regulate DDR gene expression through multiple mechanisms including epigenetic modifications [6]. We selected homologous recombination (HR) genes as a ‘target’ DDR gene subset upon which to build mechanistic insight into a possible transcriptional model for ENZ-PARP inhibitor interactions and combination therapy for CRPC, since HR genes are highly represented in upregulated DDR genes in CRPC [5], and ENZ downregulates a subset of HR genes. One of our goals was to induce AR inhibitor—mediated HR deficiency, i.e., ‘BRCAness’, and to exploit this condition to generate pharmaceutical synthetic lethality with application of a PARP inhibitor.

**Figure 1:**
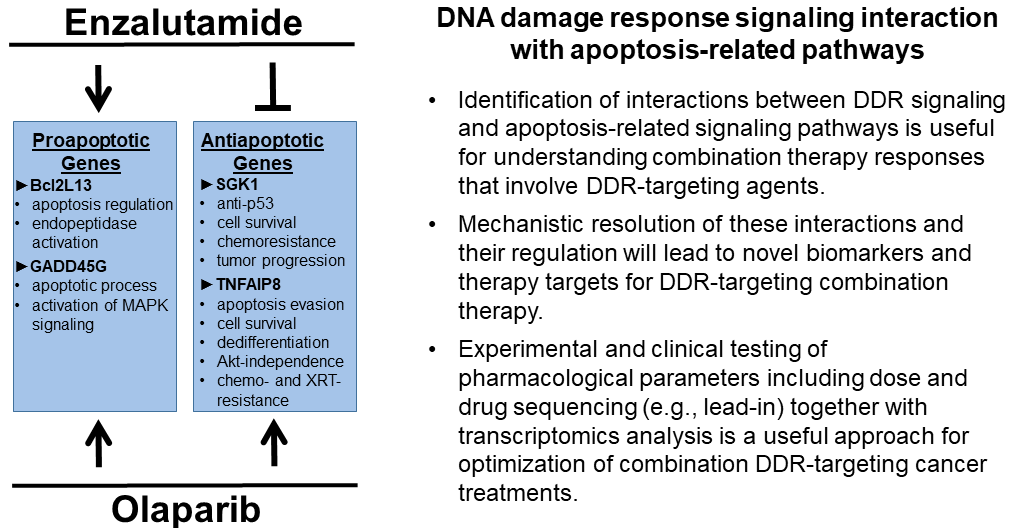
Interactions between DDR signaling and apoptosis-related pathways underlie therapy responses to DDR-targeting therapy Recent studies have shown that enzalutamide-induced BRCAness and PARP inhibition are synthetically lethal in experimental CRPC models [7]. These studies provide a template for future research that aims to identify optimized pharmacological parameters (e.g., dose and schedule) and to establish interactions between DDR signaling and apoptosis-related pathways using transcriptomics analysis of cancer cell and tissue samples following combination treatments with DDR-targeting agents. Bcl2L13, B-cell lymphoma 2-like 13; GADD45G, growth arrest and DNA damage inducible gamma; SGK1, serum/glucocorticoid-regulated kinase 1; TNFAIP8, tumor necrosis factor-alpha—induced protein 8.

We initially identified a subset of 10 HR genes which were upregulated in CRPC in 2 independent public data sets, and showed that 7 of these upregulated genes were contained within a 15 HR gene subset which was downregulated by ENZ in microarray analysis [7]. Five HR genes that were downregulated by ENZ in microarray analysis were further selected, and it was shown that ENZ suppressed these HR proteins, and that siRNA-mediated gene silencing of these genes synergized with OLA to increase cytotoxicity in PCa cells. Importantly, we further demonstrated that ENZ and OLA combination treatment led to reduced RAD51/γH2AX ratio compared to OLA treatment alone, and that ENZ and OLA combination treatment inhibited HR efficiency and induced apoptosis to a greater extent than ENZ or OLA did in PCa models *in vitro* [7]. During the course of these experiments we found that a lead-in ENZ+OLA treatment, i.e., ENZ pretreatment followed by OLA, that allowed for ENZ-mediated downregulation of HR gene prior to OLA treatment, significantly increased PCa cell apoptosis and significantly decreased colony formation compared to concomitant ENZ+OLA treatment *in vitro*. We used multiple subcutaneous and orthotopic *in vivo* CRPC models, including an ENZ-responsive PDX model to validate the superiority of lead-in combination ENZ+OLA treatment compared to concomitant administration [7]. In addition to potentially informing clinical applications of this drug combination, this finding provided an experimental tool to understand ENZ and OLA interactions at the level of gene expression within the context of apoptotic response. Our microarray data set allowed us to identify ENZ, OLA, and ENZ+OLA regulated proapoptotic or antiapoptotic genes that were aligned or antagonistic with regard to drug response. We identified multiple genes in all categories, and selected 2 ENZ and OLA upregulated proapoptotic genes, i.e., Bcl2L13 and GADD45G, and 2 ENZ-down, OLA up, antiapoptotic genes, i.e., SGK1 and TNFAIP8, to analyze by biochemical and biological assays (Figure 1). The results showed that concomitant or lead-in ENZ+OLA treatment led to an additive increase in GADD45G protein levels compared to ENZ or OLA alone. However, whereas lead-in ENZ+OLA treatment led to higher levels of Bcl2L13 protein compared to ENZ or OLA alone, concomitant ENZ+OLA did not. The results suggested that additive and potentially synergistic effects of ENZ and OLA proapoptotic activities were mediated, in part, by co-induction of target proapoptotic gene activities, and further suggested a molecular basis for the superiority of lead-in ENZ+OLA treatment. Interestingly, our results also showed that lead-in or concomitant ENZ+OLA treatment suppressed SGK1 protein to similar levels as ENZ, mitigating the inductive effects of OLA on SGK1 protein levels. In contrast, lead-in ENZ+OLA treatment demonstrated marked suppression of TNFAIP8 protein to similar levels as ENZ, whereas concomitant ENZ+OLA treatment resulted in control levels of TNFAIP8 protein, not ENZ-suppressed levels [7]. These results provided mechanistic support for the lead-in effect and further suggested that transcriptomics analysis may yield mechanistic insight into the interacting molecular pathways that underlie additive or synergistic ENZ+OLA proapoptotic therapy effects in PCa.

The causative mechanisms for susceptibility of cancers with BRCA mutations to PARP inhibitor — mediated synthetic lethality are complex, and recent studies have pointed to the need to expand the concept of BRCAness in tumors beyond germline BRCA1 or BRCA2 mutations to include mutations in genes that modulate HR and confer PARP sensitivity [8]. Our recent study which shows ENZ-induced BRCAness and PARP inhibition are synthetically lethal in experimental CRPC models suggests that further mechanistic studies of pharmaceutically induced BRCAness should be pursued [7]. Experimental and clinical testing of pharmacological parameters (including dose and drug sequencing) using experimental and clinical cell and tissue samples together with integrated genomics and transcriptomics analysis of DDR signaling and apoptosis-related pathways may lead to identification of novel biomarkers and novel therapy approaches for DDR-targeting agents.
